# Hormonal Status and the Probable Role of Phytohormones in Response of Pea Cultivar Sparkle and Mutant E107 (*brz*) to Aluminum and Iron Toxicity

**DOI:** 10.3390/plants15071129

**Published:** 2026-04-07

**Authors:** Oleg S. Yuzikhin, Alexander I. Shaposhnikov, Tatiana S. Azarova, Polina V. Guro, Miroslav I. Lebedinskii, Edgar A. Sekste, Nadezhda A. Vishnevskaya, Vera I. Safronova, Andrey A. Belimov

**Affiliations:** All-Russia Research Institute for Agricultural Microbiology, Saint-Petersburg 196608, Russia; yuzikhin@gmail.com (O.S.Y.); ai.shaposhnikov@arriam.ru (A.I.S.); tatjana-aza@yandex.ru (T.S.A.); guro.pv@arriam.ru (P.V.G.); mi.lebedinskiy@arriam.ru (M.I.L.); ea.sekste@arriam.ru (E.A.S.); na.vishnevskaya@arriam.ru (N.A.V.); vi.safronova@arriam.ru (V.I.S.)

**Keywords:** aluminum, *Pisum sativum*, iron, mutant, phytohormones, toxicity, accumulation

## Abstract

Toxic aluminum (Al) and iron (Fe) alter the hormonal balance of plants, leading to metabolic disorders and growth inhibition. Plants adapt to abiotic stress by optimizing phytohormone biosynthesis. However, the impact of toxic Al and Fe on plant hormonal status is poorly understood. Pea cultivar Sparkle and its mutant E107 (*brz*), accumulating Al and Fe due to disfunction of metal transporter gene *OPT3*, were cultivated in hydroponics supplemented or not with 80 µM of AlCl_3_ or 300 µM of FeCl_3_. Root and shoot biomass of E107 decreased due to Al or Fe treatments approximately by 30%, whereas growth of Sparkle was not affected. The Al and Fe content in the roots and shoots of the metal-treated mutant was circa twice that of Sparkle. Treatment with Al and Fe reduced the content of nutrients (Ca, K, Mg, S) in roots and/or shoots in both genotypes. Compared with Sparkle, untreated E107 possessed lower IAA and higher ethylene and tZR contents in roots but lower GA3, DHZ and tZ content in shoots. Mutant E107 had: lower GA3 and ethylene but higher DHZ, tZ and tZR contents in Al-treated roots; higher ABA, SA, IAA, GA3, DHZ, and tZ contents in Al-treated shoots; lower ABA and SA but higher JA, GA3, DHZ and ethylene contents in Fe-treated roots; higher ABA, SA, IAA, GA3, DHZ, and tZ contents in Al-treated shoots; higher ABA, JA, and GA3 but lower ethylene and tZR contents in Fe-treated shoots. Metal toxicity mainly reduced the content of phytohormones in roots and increased it in shoots. Hormonal disturbances were more significant in E107 than in Sparkle, and the effect of Al was stronger than Fe. Thus, toxic Al and Fe lead to complex, metal- and organ-specific changes in the hormonal status of E107. Hormonal changes might be associated with both defense reactions and the toxic effects of metals on plants.

## 1. Introduction

Acidic soils and soils with low redox potential contain high concentrations of mobile and plant-available forms of aluminum (Al) and iron (Fe), which are the main factors of toxicity to plants [1,2,3,4]. In response to the excess of these elements in the soil, plants have developed a number of resistance mechanisms that have common features: increasing the rhizosphere pH, complexation by root exudates, production of mucilage and siderophores, detoxification within root tissues, exclusion from roots, storage in unavailable forms, and metabolic tissue tolerance processes [2,3,5,6,7]. Aluminum is a non-essential metal for plant growth, whereas Fe is a very important micronutrient involved in many physiological processes [8]. Accordingly, studies have focused on the negative effects and protective mechanisms of plants against Al toxicity, while with regard to Fe, the emphasis has been on studying the mechanisms of homeostasis, uptake, transport, and avoidance of deficiency of this microelement [3,9,10,11].

Abiotic stresses significantly affect biosynthesis and action of phytohormones and vice versa. These signal compounds play an important role in mitigating or even strengthening the negative effects of unfavorable environment on plants [12]. The hormonal response of plants to Al toxicity was studied mainly for auxins, abscisic acid (ABA), and ethylene [13]. Examples of the Al effects on concentrations of various phytohormones in plants are collected in Table S1. All cited studies were carried out under hydroponic culture conditions, which allowed isolating the effect of the stressor from the accompanying factors presented in soil, and the plants were subjected to toxic Al in relatively short periods of time, measured in hours (Table S1). As a rule, Al toxicity resulted in increased levels of ethylene, ABA, and salicylic acid (SA), which are phytohormones that are indicators of stress response in various plant species. Opposite effects of Al on the content of indole-3-acetic acid (IAA), ranging from an increase [14,15,16,17,18,19] to decrease [20,21,22], were described. Limited information is available about the changes in cytokinin (CK) levels under the influence of Al treatment on plants. Namely, increased content of zeatin (Z), zeatin riboside (ZR), dihydrozeatin riboside (DHZR), and zeatin riboside-5-monophosphate (ZMP) in roots of the common bean (*Phaseolus vulgaris* L.) was only reported [23,24]. It should be noted that little is known about hormonal response of pea (*Pisum sativum* L.) to Al toxicity.

Involvement of phytohormones in the uptake, translocation, and regulation of Fe nutrition under optimal and Fe-deficient growth conditions is substantial and is well studied [9,10,11]. However, limited information is available on the effects of toxic Fe concentrations on these signal molecules in plants. Ethylene, auxins, and nitric oxide (NO), as well as their crosstalk, have been shown to play a role in plant responses to this stress [3,7]. Particularly, toxic Fe increased IAA and ABA in roots of Fe-sensitive and Fe-tolerant wheat cultivars [25]. Treatment with Fe_2_O_3_ nanoparticles decreased IAA and ABA in roots of rice, but it had no effect on hormone content in shoots [26]. Increased ethylene release by Fe-treated *A. thaliana* roots was combined with elevated Fe tolerance of ethylene overproducing mutants *eto1-1* and *eto2-1* [27]. Rice responded to Fe toxicity by the increased ethylene production in roots and leaves [28,29]. The impact of excess Fe on the complex and balance of phytohormones remains poorly understood.

The pleiotropic pea mutant E107 (*brz*) was obtained using variety Sparkle by treatment with ethyl methanesulfonate and showed decreased ability to form symbiotic nodules with rhizobia [30,31], decreased biomass and lateral root number [31,32], necrotic spots on leaves, and increased concentrations of Fe [30,33], Ca, K, Mg [30,33], Cu, Zn, and Pb [34] in shoots. Shoot Al content was about 12 times higher in E107 mutant grown in vermiculite [32] or in soil [34] and toxicity symptoms on the mutant roots were observed via hematoxylin staining [32]. Increased Al content in roots and inhibition of biomass production was also observed when E107 was cultivated in Al supplemented hydroponics, suggesting lower tolerance of the mutant to Al [35]. Active acidification of the rhizosphere due to release of protons [32,33] and constitutively increased Fe(III) reductase activity [36] of E107 mutant suggested Fe-deficient phenotypes under optimum Fe supply. Increased concentrations of organic acids amino acids and sugars were detected in root exudates of E107 that also could contribute to active mobilization and uptake of nutrient and nonessential elements [35].

The single recessive gene, associated with decreased nodulation, Fe accumulation, and bronze leaf spots of E107, was designated *brz* (bronze) [30], and for a long time, this gene was not identified. Recently, it was found that the *brz* phenotype is associated with mutations in gene *OPT3*, encoding an oligopeptide transporter with a plant-specific role in transport of various metals [37]. Experiments with *A. thaliana* knockout mutants showed that *OPT3* controlled Fe transport from roots to leaves [38,39], as well as Fe, Mn, and Zn transport from xylem to phloem [40]. Overexpression of *OPT3* resulted in accumulation of Cd in *A. thaliana* seeds [39,40]. Response of *A. thaliana* to Fe-deficiency showed that *OPT3* could be regulated by ethylene [41,42]. Treatment of E107 with ethylene inhibitors aminoethoxyvinylglycine (AVG) and Ag^+^ ions restored nodulation [31], whereas aminooxyacetic acid (AOA) and Co^2+^ ions inhibited Fe(III) reductase activity and decreased Fe content in shoots and stimulated growth [43]. These observations also suggested involvement of ethylene in phenotype manifestation of the mutant. However, information about the role of *OPT*3 in hormonal status and response of plants to Al and Fe toxicity is limited. Little is also known about the effect of these metals on phytohormones of pea variety Sparkle and its E107 mutant.

The aim of the present report was to characterize differences between wild type variety Sparkle and mutant E107 in hormonal responses to Al and Fe toxicity for better understanding the role of metal transporters like OPT3 in accumulation of these metals by plants and in modulation of plant phytohormone status caused by toxic metals.

## 2. Results

**Figure 1:**
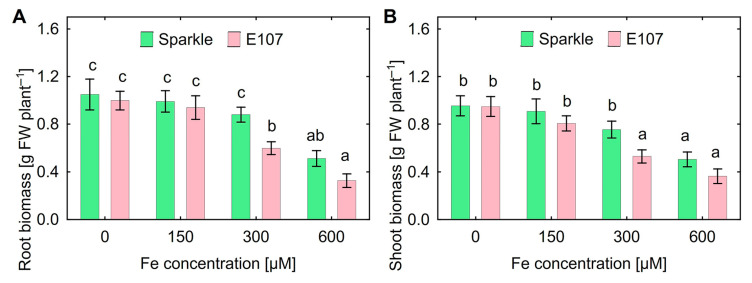
Root (**A**) and shoot (**B**) growth response of cultivar Sparkle and E107 to different concentrations of FeCl_3_ supplemented to the nutrient solution. Means and SE of one experiment. Different letters indicate significant differences between genotypes and treatments (Fisher’s LSD test, *p* < 0.05, n = 6). Sparkle is shown as the left column and E107 as the right column in both parts of the figure.

An experiment to select the appropriate Fe concentration showed that inhibition of root and shoot growth occurred at 300 µM of FeCl_3_ for E107 mutant and at 600 µM of FeCl_3_ for Sparkle (Figure 1). Significant differences in the effect of Fe on plant biomass between genotypes were observed at 300 µM FeCl_3_. Therefore, a concentration of 300 µM of FeCl_3_ was chosen for subsequent experiments.

**Figure 2:**
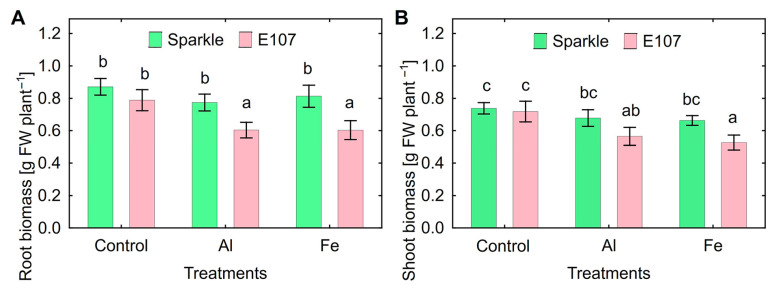
Root (**A**) and shoot (**B**) growth response of cultivar Sparkle and mutant E107 to toxic concentrations of Al and Fe supplemented to the nutrient solution. Treatments: Control—untreated plants; Al—80 µM of AlCl_3_; Fe—300 µM of FeCl_3_. Means and SE of two experiments. Different letters indicate significant differences between genotypes and treatments (Fisher’s LSD test, *p* < 0.05, n = 12). Sparkle is shown as the left column and E107 as the right column in both parts of the figure.

Treatments with the chosen Al and Fe concentrations did not affect biomass of roots (Figure 2A) and shoots (Figure 2B) of Sparkle. Root and shoot biomass of E107 decreased by Al and Fe treatments to approximately the same extent (Figure 2). Content of Al in roots and shoots of E107 was about twice as compared with Sparkle (Table 1). Mutant E107 had increased Fe content in roots and shoots in the presence and absence of metals. Particularly, mutant’s roots and shoots possessed, respectively, 53% and 120% more Fe content then Sparkle in the presence of toxic Fe (Table 1).

Treatment with Al decreased Ca content in roots of E107 (Table 2). Treatments with Al and Fe decreased K, Mg, and S in roots (Table 2) and Ca and Mg in shoots (Table 3) of both pea genotypes. Fe toxicity lead to a decrease in root Mn and shoot S and Zn contents of mutant E107. The untreated mutant E107 had higher Mn and Zn content in roots and Ca and Mn content in shoots.

**Figure 3:**
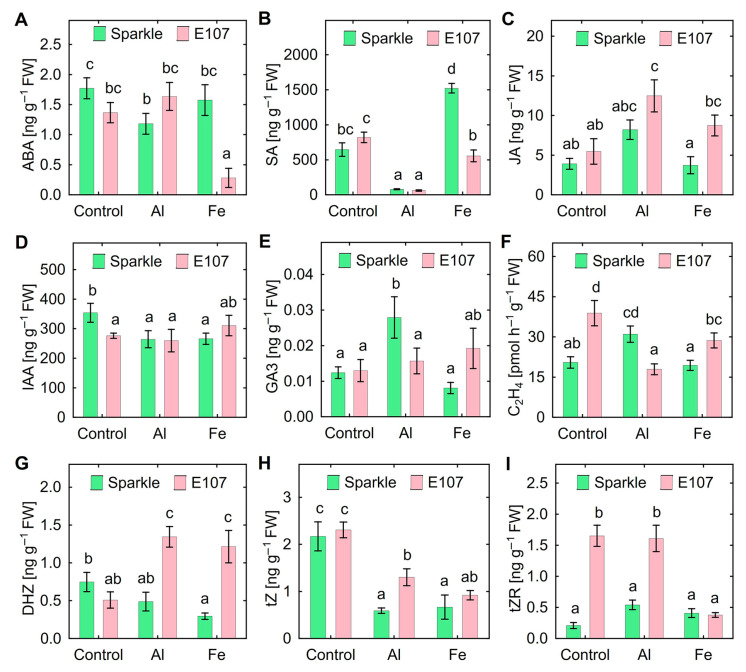
Content of phytohormones ABA (**A**), SA (**B**), JA (**C**), IAA (**D**), GA3 (**E**), C_2_H_2_ (**F**), DHZ (**G**), tZ (**H**) and tZR (**I**) in roots of cultivar Sparkle and mutant E107 treated with toxic concentrations of Al and Fe. Treatments: Control—untreated plants; Al—80 µM of AlCl_3_; Fe—300 µM of FeCl_3_. Means and SE of two experiments. Different letters indicate significant differences between genotypes and treatments (Fisher’s LSD test, *p* < 0.05, n = 6). Abbreviations: ABA—abscisic acid; SA—salicylic acid; JA– jasmonic acid; IAA—indole-3-acetic acid; GA3—gibberellic acid; DHZ—dihydrozeatin; tZ—trans-zeatin; tZR—trans-zeatin riboside.

**Figure 4:**
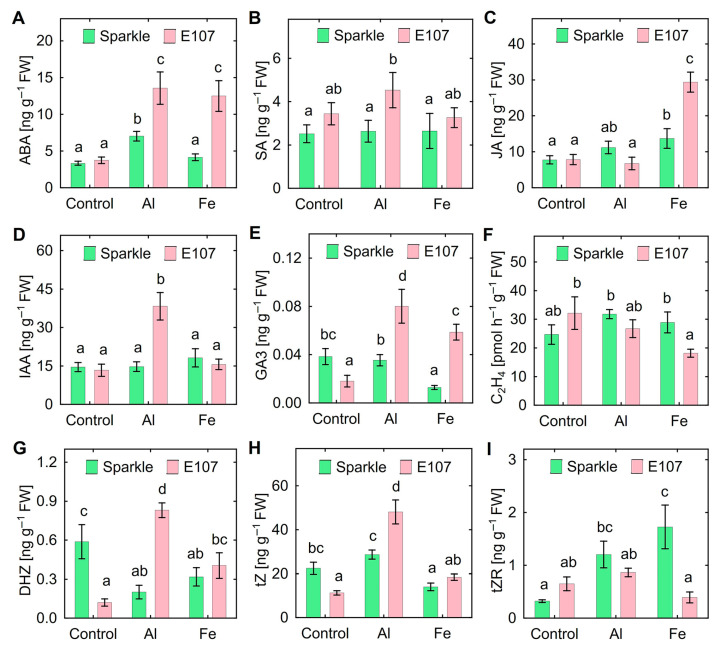
Content of phytohormones ABA (**A**), SA (**B**), JA (**C**), IAA (**D**), GA3 (**E**), C_2_H_2_ (**F**), DHZ (**G**), tZ (**H**) and tZR (**I**) in shoots of cultivar Sparkle and mutant E107 treated with toxic concentrations of Al and Fe. Treatments: Control—untreated plants; Al—80 µM of AlCl_3_; Fe—300 µM of FeCl_3_. Means and SE of two experiments. Different letters indicate significant differences between genotypes and treatments (Fisher’s LSD test, *p* < 0.05, n = 6). Sparkle is shown as the left column and E107 as the right column in all parts of the figure. Abbreviations are the same as in Figure 3.

In the absence of toxic Al and Fe, mutant E107 possessed lower content of IAA (Figure 3D) and higher content of ethylene (Figure 3F) and *trans*-zeatin-ribozide (tZR) (Figure 3I) in roots. Shoots of the untreated E107 mutant showed decreased gibberellic acid (GA3) (Figure 4E), dihydrozeatin (DHZ) (Figure 4G), and *trans*-zeatin (tZ) (Figure 4H) content.

Treatment with Al decreased ABA, SA, IAA, and tZ content but increased GA3 content and ethylene production in roots of Sparkle (Figure 3). The Al-treated mutant roots had decreased SA, ethylene, and tZ content but increased jasmonic acid (JA) and DHZ content (Figure 3). Treatment with Fe decreased IAA, DHZ, and tZ content but increased SA content in roots of Sparkle (Figure 3). The Fe-treated mutant roots had increased DHZ content but decreased ABA, SA, tZ, and tZR content and ethylene production (Figure 3). In the presence of toxic metals, the genotypic differences in the root phytohormone content were as follows: (1) lower ethylene production but higher content of DHZ, tZ, and tZR content in Al-treated roots; (2) lower ABA and SA content but higher JA, GA3, and DHZ content and ethylene production in Fe-treated roots (Figure 3).

Differences between genotypes in shoot phytohormone concentrations in metal untreated plants were lower GA3, DHZ, and tZ contents in the mutant (Figure 4E,G,H). Treatment with Al increased ABA and tZR content but decreased DHZ content in shoots of Sparkle (Figure 4). Mutant E107 responded to Al treatment by increased ABA, IAA, GA3, DHZ, and tZ content in shoots (Figure 4). Treatment with Fe decreased GA3, DHZ, and tZ content but increased JA and tZR content in shoots of Sparkle (Figure 4). Mutant E107 responded to Fe treatment by decreased ethylene production and increased ABA, JA, GA3, and DHZ content (Figure 4). Genotypic differences in Al-treated shoots were expressed as higher ABA, SA, IAA, GA3, DHZ, and tZ content in the E107 mutant (Figure 4). The Fe-treated mutant shoots had elevated ABA, JA, and GA3 content but lower ethylene production and tZR content (Figure 4).

**Figure 5:**
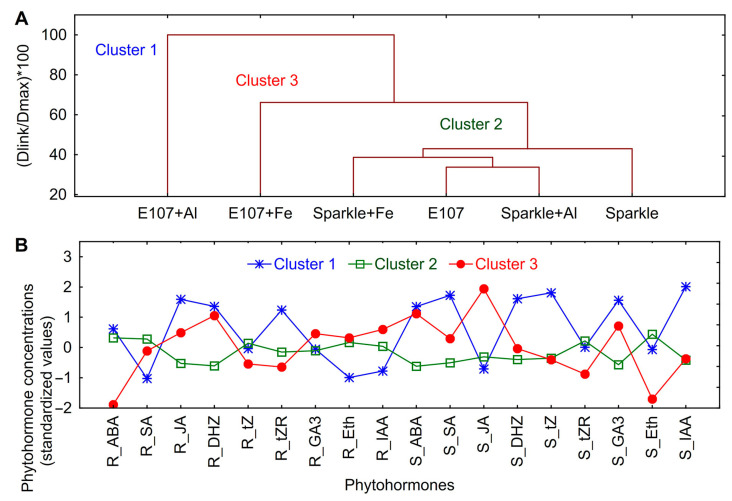
Cluster dendrogram (**A**) and plot of means for each cluster (**B**) showing the relationships among genotypes and treatments based on the data for contents of phytohormones in cultivar Sparkle and mutant E107. Treatments: Al—80 µM of AlCl_3_; Fe—300 µM of FeCl_3_. R stands for roots and S stands for shoots in *X*-axis caption of part B. Standardized values, Ward’s method for linkage rules, squared Euclidian distance. Abbreviations are the same as in Figure 3.

Cluster analysis of the data for phytohormone contents showed that all Sparkle variants and the control E107 variant were grouped into a homogeneous cluster 2 at a similarity level of approximately 40% (Figure 5A). Variants of E107 mutant treated with Al and Fe formed separate clusters 1 and 3, respectively. Variants in cluster 2 were characterized by relatively low variability in standardized values of phytohormone content (Figure 5B). Variants E107 treated with Al or Fe had high variability and the opposite deviation from cluster 2 for several hormones. The phytohormones that caused the grouping of metal-treated E107 variants into separate clusters were identified and shown in Figure 4B. Among them, the maximum difference of Al-treated E107 from cluster 2 was for JA, DHZ, ethylene, and IAA, both in roots and shoots. The maximum difference of Fe-treated E107 from cluster 2 was for ABA and JA, both in roots and shoots, as well as root DHZ and shoot ethylene (Figure 5B). The maximum and opposite differences between E107 treated with Al and treated with Fe were in JA, DHZ, ethylene, and IAA both in roots and shoots, as well as in root ABA and tZR and shoot SA and tZ (Figure 5B).

## 3. Discussion

### 3.1. Plant Growth and Uptake of Elements

Reduced resistance of the E107 mutant to high Al concentrations expressed as growth inhibition as compared to Sparkle was shown previously [35] and corresponds with the present report (Figure 2). Comparison of E107 with Sparkle in response to Fe toxicity was not previously estimated, and this study showed decreased Fe tolerance of E107 in root and shoot biomass production (Figure 1 and Figure 2). It is most likely that such a response is associated with the active accumulation of Al and Fe by the mutant (Table 1).

Increased accumulation of Al [31,33,35], Fe [30,31,32,37,43], and various nutrient elements [31,33,34,35] in shoots of E107 mutant has been repeatedly described. It should be noted that in these publications, the concentrations and type of the measured element affected in the mutant shoots varied significantly depending on growing conditions, substrate for plant cultivation, plant age, and treatments with chemicals. In the presented experiments, an increased content of elements was also observed in E107, and the results are in good agreement with our previously obtained data on the elemental composition of the Al-treated mutant grown in roughly similar conditions [35].

Similarity between Sparkle and E107 in the response to the toxicity of both Al and Fe was expressed in a decrease in the content of nutrients (Table 2 and Table 3). Such similarity pointed to insignificant role of the mutated *OPT3* gene in the accumulation of nutrients, except Fe, by the plants subjected to Al or Fe toxicity. The only exception was the increased Mn content in the shoots of E107 treated with Al, but the explanation of this effect requires more detailed study. Interestingly, knock-out *OPT3* mutant of *A. thaliana* decreased accumulation of toxic Cd in shoots [40], whereas overexpression of *OPT3* increased Cd accumulation in seeds [39]. Mutant E107 had four-fold higher Pb content in shoots as compared with Sparkle when plants were grown in contaminated soil [34]. Thus, more attention should be given to the study of the role of the *OPT3* gene in the accumulation of biogenic and abiogenic elements and convenient model for this may be the E107 mutant.

### 3.2. Auxins

Reduction in IAA content in metal-untreated E107 roots (Figure 3D) is an interesting finding for characterizing this mutant and the role of the *OPT3* in hormonal balance of plants. However, the significance of this observation for the root response to toxic metals may be questionable, since both genotypes had similar IAA content in Al- and Fe-treated roots (Figure 3D). Auxins are involved in plant responses to Al toxicity, but their role is ambiguous [13,44]. Treatment with exogenous IAA weakened the Al-induced inhibition of maize root elongation [20], reduced Al content in alfalfa root tips by increasing rhizosphere pH and weakening Al binding by pectin in root tips [22], and stimulated efflux of malate by wheat roots [15]. Overexpression of auxin transporter *OsPIN2* decreased oxidative stress, lignification, and accumulation of Al in cell wall of rice roots due to active Al compartmentation in vacuoles [16,45]. Rice mutant with knock-down of the auxin carrier gene *OsAUX3* showed lower Al content and Al-induced oxidative damage in roots [18]. These findings suggested positive regulation Al tolerance by auxins. On the other hand, decreased Al tolerance of *A. thaliana* auxin-overproducing mutant *yucca* and of wild type plants treated with auxin analog naphthylacetic acid were demonstrated [46].

Shoots of E107 responded to Al treatment by 2.5 times increase in IAA content (Figure 4D). This effect is difficult to explain, as there is no information on the effect of Al on IAA content in plant shoots. Given the positive regulation of Al resistance in roots by auxins [15,16,18,20,22,45], it can be assumed that this is an activation of defense mechanisms in the genotype having high toxicant accumulation.

Decreased IAA content in Sparkle roots treated with either Al or Fe (Figure 3D) is in line with decreased IAA content in rice roots treated with Fe_2_O_3_ nanoparticles [26], but it contradicts the data on the increase in the content of this hormone in wheat roots in the presence of toxic Fe [25]. Auxins increase Fe(III) reductase activity during Fe deficiency [47,48], and a decrease in their content might be due to excess Fe in plants with normal Fe homeostasis. However, this mechanism does not function in the E107 mutant with constitutive Fe deficiency [36].

### 3.3. Ethylene

Ethylene evolution by roots of Sparkle in the amount of 23 pmol h^−1^ g^−1^ FW was previously reported [49], and this corresponds well with our results (Figure 3F). It was found that Fe deficiency increased ethylene evolution by Sparkle roots and treatment with ethylene inhibitor, 1-aminocyclopropane-1-carboxylic acid (ACC), stimulated its Fe-reducing capacity [50]. Under sufficient Fe supply, treatments of E107 with ethylene inhibitors aminooxyacetic acid (AOA) and Co^2+^ ions inhibited Fe(III) reductase activity, decreased Fe content in shoots, and stimulated plant growth [43].

Exposure of *Lotus japonicus* [51] and *A. thaliana* [52] (Sun et al., 2010) to toxic Al elicited a rapid ethylene evolution and inhibited root elongation. Root elongation in the ethylene-signaling deficient mutants *etr1-3* and *ein2-1* was less inhibited than that in wild-type *A. thaliana* plants, whereas ethylene inhibitors Co^2+^ and aminoethoxyvinylglycine (AVG), eliminated the Al-induced inhibition of root elongation [52]. However, overexpression of *GsERF1* gene from Al-resistant *Glycine soja* line BW69 in *A. thaliana*-stimulated root growth, weakened the hematoxylin staining of hairy roots, and upregulated the ethylene synthesis-related genes (*ACS4*, *ACS5,* and *ACS6*) and stress/ABA-responsive marker genes (*ABI1*, *ABI2*, *ABI4*, *ABI5,* and *RD29B*), suggesting enhanced Al tolerance through an ethylene-mediated pathway and/or ABA-signaling pathway [53]. Thus, in most reports, the results suggested negative regulation of Al tolerance by ethylene.

In the metal untreated plants, mutant E107 showed about two times increased ethylene production by roots as compared with Sparkle (Figure 3F), indicating the relationship between the functioning of the *OPT3* gene and the biosynthesis of this hormone. Such relationship was shown using the *A. thaliana* mutant *opt3-2*, which contained more ACC and higher expression of ethylene synthesis and signaling genes in roots [41]. Interestingly, elevated production of ethylene by E107 roots observed here (Figure 3F) could be responsible for the reduced nodulation frequency of this mutant reported previously [30,31]. However, little is known about the involvement of *OPT3* in ethylene production by plants subjecting to Al stress. Decreased ethylene production by Al-treated E107 mutant (Figure 3F) was probably due to the toxic effect of accumulated Al causing oppression of plant metabolism, since regulatory inhibition of ethylene by Al is unlikely according to the literature. The increase in ethylene content in Al-treated Sparkle roots (Figure 3F) is consistent with the literature data related to the effect of Al toxicity on other plant species [24,51,52,54]. Here, an important genotypic difference was the opposite response to Al toxicity in root ethylene production.

Ethylene is involved in modulation of Fe(III) reductase activity and controls Fe uptake and homeostasis under deficiency of this micronutrient [55]. Toxic Fe concentrations stimulated ethylene production by rice [28,29] and *A. thaliana* [27] roots. Treatment of *A. thaliana* with AVG strengthened inhibition of lateral root development in the presence of toxic Fe, whereas wild-type plants treated with ACC and ethylene-overproducing mutants *eto1-1* and *eto2-1* showed enhanced Fe tolerance [27,56]. Ethylene, being an antagonist to NO that inhibited root growth of *A. thaliana* and exerted stimulating effect on roots treated with Fe [57]. These findings demonstrated positive role of ethylene in plant tolerance to Fe toxicity.

Thus, ethylene possessed the opposite effect on plant tolerance to Al and Fe. This may likely explain the differences in ethylene production by roots (Figure 3F) or shoots (Figure 4F) observed in plants treated with Al and Fe. The ethylene response pattern becomes more complex when comparing genotypes differ in the tolerance and accumulation of these metals. Genotypic differences between Sparkle and E107 emphasized the role of the *OPT3* gene in ethylene production under normal and stressed conditions.

### 3.4. Abscisic Acid

Similar ABA content in Sparkle and E107 in the absence of toxic metals (Figure 3A and Figure 4A) suggested little effect of *OPT3* gene on ABA metabolism under normal nutrient conditions. However, genotypic differences were evident in the presence of Al and Fe both in roots and shoots. Literary data indicate increased ABA content in Al-treated roots of pea and maize [58], rice [59], barley [58,60], soybean [61], and *A. thaliana* [62]. ABA plays a positive role in Al tolerance due to increasing expression of ALMT genes, stimulating exudation of organic acids and biosynthesis of antioxidants [44]. The discrepancy between our results and these data might be due to the different duration of plant exposure to the toxicants. Namely, the exposure in the cited studies ranged from 3 to 72 h (Table S1), while our experiments lasted 10 days and the effect can be characterized as chronic.

The effect of Fe toxicity on ABA content in plants has been little studied. With excess Fe, the action of ABA was aimed at reducing its accumulation in *A. thaliana* seeds, but with Fe deficiency, the effect was the opposite [63]. Toxicity of Fe caused an increase in ABA content in wheat roots treated for 4 days with 15 mM of Fe-EDTA [25], whereas decreased root ABA content was observed in rice treated for 7 days with 20 or 200 mg L^−1^ of Fe_2_O_3_ nanoparticles [26]. These results are difficult to compare, but they are consistent with the hypothesis of increasing ABA levels as a rapid defense response to toxicity and decreasing ABA levels caused by metabolism disturbance as a result of chronic exposure. The high content of ABA in shoots was probably due to the later reaction of shoots compared to roots due to the sequential entry of toxicants through plant organs. In this regard, the more active ABA accumulation in the mutant shoots (Figure 4A) could be due to the higher content of toxic Al and Fe (Table 1) as compared with Sparkle. Thus, disruption of the toxic metal transport control caused by mutation in *OPT3* gene led to active accumulation of ABA in the shoots of the E107 mutant.

### 3.5. Cytokinins

The effect of toxic Al and Fe on CK and the role of CK in the resistance and accumulation of these metals by plants are poorly studied [44]. AI-induced increase in content of cytokinin-like substances in the roots of spruce (*Picea abies* L.) seedlings grown in hydroponics was observed [14]. Increased content of Z, ZR, and DHZR was found in Al-treated bean roots [23,24]. Application of exogenous synthetic CK 6-benzylaminopurine (BA) stimulated lateral shoot growth and branching and uptake of nutrients Ca, K, and Mg by soybean [64]. In contrast, treatment with BA enhanced Al-induced root growth inhibition in *A. thaliana* [65]. Elevated content of CK could be involved in root growth inhibition by regulation of apical meristem and crosstalk with other phytohormones [66]. Particularly, exogenous BA and Z inhibited root elongation and stimulated IAA and ethylene biosynthesis in pea roots [67]. In our experiments, Al-treated mutant E107 had a two- or three-fold increase in the content of several CK in roots and shoots, as compared with Sparkle. It could be suggested that CK was involved in the reduced resistance of the E107 mutant to Al. However, plant biomass did not correlate with the CK contents, with the exception of a negative correlation (r = −0.83, *p* = 0.040, n = 6) between root biomass and the DHZ content in roots (Table S2).

The differences in CK contents between Sparkle and E107 in plants treated with Fe were less pronounced than in those treated with Al. Therefore, linking this parameter to plant resistance to Fe appears difficult. The only report we know of on this topic described reduction of Fe-uptake and oxidative stress, increase in chlorophyll content and grain yield of rice cultivated in ferric soil and sprayed with kinetin [68].

### 3.6. Salicylic Acid

SA is an important signaling molecule, mediating plant responses to abiotic stresses, particularly counteracting heavy metal toxicity largely due to the strengthening of antioxidant systems [44,69,70]. However, in this area of knowledge [3,10,71], little attention has been paid to studying the role of SA in plant resistance to Al and Fe. Toxic Al concentrations increased SA content in roots of *Cassia tora* [72] and soybean [73,74]. Treatment with SA-stimulated citrate efflux by *Cassia tora* [72] and soybean roots [73,74] and increased expression of *GmMATE1* gene [73], leading to a reduction in the Al content in the roots and improved growth. Hydroponically grown mutant E107 exuded three-fold more SA than Sparkle. However, toxic Al decreased exudation of both genotypes by 10-fold and eliminated genotypic differences [35]. This result is in good agreement with the negative effect of Al on the SA content in roots of both pea genotypes found in the present study (Figure 3B). Treatment with Al caused approximately the same change in the content of SA in Sparkle and E107 (Figure 3B and Figure 4B). Therefore, the influence of the *OPT3* gene on this phytohormone in Al-treated roots or shoots seems insignificant. Importantly, the *OPT3* gene might be responsible not only for increased proton release [33], but also for the exudation of a large complex of organic low-molecular weight compounds (organic acids, amino acids, and sugars), as was described previously in E107 [35]. The difference between E107 and Sparkle in root exudation of SA and other compounds requires further research. Activation of protective antioxidant systems by exogenous SA was described for pea to reduce the toxicity of B [75], Cd [76], Cr [77], Pb [78], and NaCl [79]. The role of SA in plant resistance to Fe toxicity also needs to be studied, as information on this issue is lacking.

### 3.7. Jasmonic Acid

The absence of genotypic differences in the JA content in the control and Al-treated E107 mutant and Sparkle (Figure 3C and Figure 4C) indicated a low probability for the involvement of *OPT3* gene in plant responses to Al toxicity associated with this phytohormone. In contrast, Fe toxicity caused a two-fold increase in the JA content in the mutant roots and shoots (Figure 3C and Figure 4C), indicating the important role of the mutation. According to recent reviews, information on the role of JA in plant resistance to Al and Fe is fragmentary and contradictory [7,44,80,81,82]. Exogenous methyl-jasmonate (MeJA) increased expression of genes related to citrate transport and Al-binding, decreased Al content in roots, and stimulated root growth of Al-treated rice [83]. Blueberry plants responded positively to treatment with low MeJA concentration (5 μM) by accumulation of phenolic compounds and increment of SOD activity resulting in reduced oxidative damage caused by Al [84]. However, high MeJA concentration (50 μM) exerted toxic effect on plants. Inhibition of root growth of Al-treated plants by exogenous JA was observed on tomatoes [85], *A. thaliana* [86], and *Cassia tora* [87]. Little is known in the involvement of JA in tolerance of pea to Al and Fe toxicity. Our experiments revealed negative correlation between root biomass and JA content in roots (r = −0.88, *p* = 0.022, n = 6) in the studied pea genotypes (Table S2). It is more likely that the reduced Al and Fe tolerance and the increased JA content in E107 mutant are interconnected and indicate a negative role of this phytohormone in stress response.

### 3.8. Gibberellins

Differences between treatments and/or genotypes in the root GA3 content were mostly insignificant due to high data variability, and the only significant effect was a 50% increase in GA3 content in Sparkle treated with Al (Figure 3E). The untreated mutant E107 had lower shoot GA3 content, but toxic Al and Fe increased GA3 content several times only in E107 (Figure 4E). This indicates the involvement of the *OPT3* gene in the response of pea shoots to the toxicity of both metals. It was previously shown that the GA-deficient rice mutant had abolished the hydrogen-enhanced Al tolerance in roots, suggesting positive role of GA in counteracting toxicity [88]. On the contrary, exogenous GA3 reduced the resistance of rice to Al, causing an increase in accumulation of reactive oxygen species and decrease in the expression of Al-tolerance genes *ART1*, *OsNramp4,* and *SAL1* [89] (Lu et al., 2024). Overexpression of the gene *SD1* for gibberellin biosynthesis reduced Al resistance of rice, whereas the mutant STR1 with impeded GA signaling had increased resistance to Al [89]. Based on this scant and contradictory information, it is difficult to interpret the obtained results, and more detailed studies are needed to determine the role of GA and the relationship of GA with the *OPT3* gene in plant resistance to Al.

### 3.9. Hormonal Balance

The clustering pattern of variants based on phytohormone content showed that Sparkle maintains homeostasis to a greater extent than the E107 mutant (Figure 5). The hormonal imbalance in E107 was likely due to the accumulation of Al and Fe to toxic concentrations. It is less likely that this was due to inhibition of nutrient uptake, since the response of both genotypes to toxicants in terms of elemental contents was similar (Table 2 and Table 3).

The relative similarity in hormonal status between the untreated Sparkle and 107 plants under normal growth conditions indicated a minor effect of the mutation in *OPT3*, which was associated with metal transport. Hormonal differences between the genotypes manifest themselves primarily under stressful conditions, and, to a greater extent, with Al treatment than with Fe. This was evidenced by the formation of a separate Cluster 1, by the aluminum-treated mutant, which is most distant from Cluster 2 grouping untreated plants (Figure 5). Differences in plant hormonal responses to Al and Fe might be due to specific stress characteristics caused by the abiotic element Al and the essential micronutrient Fe.

**Figure 6:**
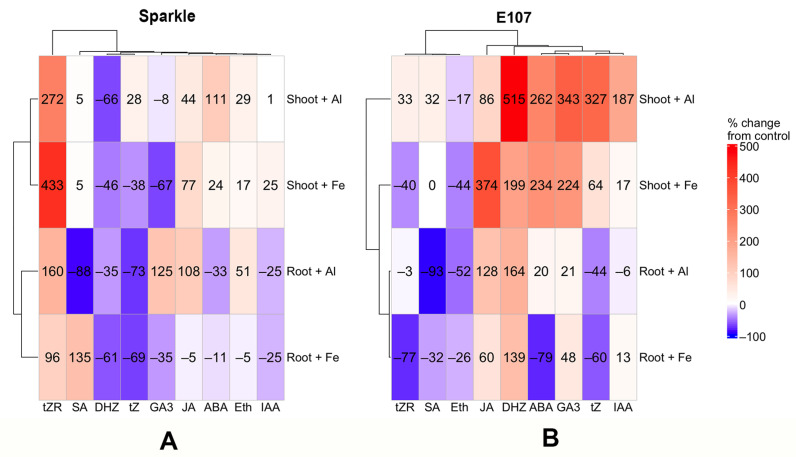
Heat maps for the effects of Al and Fe on phytohormone contents in cultivar Sparkle (**A**) and mutant E107 (**B**). Hierarchical clustering was conducted using Ward’s method (ward.D) based on squared Euclidean distances. Color intensity represents the percent change from control values, where 0% corresponds to the untreated control. Abbreviations are the same as in Figure 3.

To visualize and simplify the perception of changes in the hormonal status of the studied pea genotypes under Al and Fe toxicity, a heat map was generated (Figure 6). The greatest imbalance in shoots was detected in E107 and manifested primarily by increased levels of most phytohormones, with the exception of TP3 and ethylene. The response to Al and Fe was similar for several phytohormones in Sparkle roots (tZR, tZ, and IAA) and shoots (DHZ, tZR, JA, and ethylene). The mutant also had this similarity, but it was manifested for the following phytohormones: in roots—ethylene, JA, DHZ, and tZ; in shoots—ethylene, JA, DHZ, ABA, GA3, and tZ (Figure 6). This indicated similarities in biochemical targets and/or defense mechanisms to the effects of these metals, possibly due to their similarity as trivalent cations. However, this speculation needs experimental verification. It should also be taken into account that both Al and Fe can be toxic to plants in acidic soils, promoting the development of common responses. On the other hand, some hormones responded oppositely to Al and Fe. Specifically, in Sparkle, there were SA and GA3 in roots and tZ in shoots. In the E107 mutant, these hormones were ABA in roots and tZR in shoots (Figure 6). This observation demonstrated the specificity of stress responses induced by Al and Fe. A more in-depth analysis of this phenomenon may help uncover the distinctive features of resistance mechanisms to these metals.

The most pronounced genotypic differences between Sparkle and E107 in the hormonal response to Al toxicity were observed in roots for tZR, DHZ, and ethylene, while in shoots the most pronounced differences were for tZ, GA3, and IAA, respectively (Figure 6). Hormonal response to Fe toxicity specific to the E107 mutant was detected for tZR, DHZ, GA3, JA, and ABA in roots, as well as for tZR, DHZ, tZ, GA3, JA, ABA, and ethylene in shoots (Figure 6). These differences from Sparkle varied in magnitude and ranged from positive to negative. The obtained results demonstrate the important role of the *OPT3* gene in regulating plant hormonal status under stressful conditions.

Importantly, the hormonal imbalance of the E107 mutant was more pronounced in shoots and was characterized by elevated phytohormone levels. Previously, it was proposed the existence of a shoot-to-root signal for iron deficiency in the E107 mutant [33,90,91]. Then, experiments with *A. thaliana* mutant *opt3-2* provided evidence that this gene is a component of the iron-signaling network between leaves and roots [39,40]. Here, we showed that misregulation of *OPT3* leads to significant disturbance of the hormonal status of plants, especially under stressful conditions. The spectrum and pattern of changes had a specific character depending on the toxic metal and plant organ. The identified genotypic differences can be useful to decipher the mechanisms of interactions between phytohormones and metal transport in plants.

The effect of Al and Fe on E107 mutant was manifested basically by a decrease in phytohormone levels in the roots that correlated poorly with the published data on increased phytohormone levels caused by Al toxicity (Table S1). This may be explained by the relatively short plant exposure to Al in most of the cited studies, which lasted several hours. Long-term exposure in our experiments could have had a toxic effect on phytohormone biosynthesis in the roots. By the end of the experiments, Al accumulated in the shoots of the E107 mutant and caused an increase in phytohormone levels (Figure 6B). Long-term exposure more closely reflects plant behavior under real soil conditions and characterizes the hormonal balance of plants better adapted to stress. Thus, more detailed studies of plant vegetation dynamics are necessary to understand hormonal changes during chronic exposure to the toxicant.

## 4. Materials and Methods

### 4.1. Plants

Seed samples of pea (*Pisum sativum* L.) cultivar Sparkle and its mutant E107 (*brz*) were kindly provided by Prof. Frederique C. Guinel. The seeds were propagated under the same soil and climate conditions in summer under natural light, air moisture, and temperature (experimental greenhouse of ARRIAM, Saint Petersburg, Russia).

### 4.2. Hydroponic Culture

The applied hydroponic gnotobiotic system was previously described in detail [92]. Briefly, seeds were disinfected with 98% H_2_SO_4_ for 20 min, followed by rinsing with sterile tap water 10 times and germination in glass Petri dishes for 3 days at 22 °C. Then, uniformly germinated seedlings were transferred to each polypropylene pot containing 1000 mL of nutrient solution (µM): KNO_3_, 1200; KH_2_PO_4_, 400; Ca(NO_3_)_2_, 60; MgSO_4_, 250; KCl, 250; CaCl_2_, 60; Fe-tartrate, 12; H_3_BO_3_, 2; MnSO_4_, 1; ZnSO_4_, 3; NaCl, 6; Na_2_MoO_4_, 0.06; CoCl_2_, 0.06; CuCl_2_, 0.06; NiCl_2_, 0.06; pH = 4.7. Nutrient solution was supplemented or not with 80 µM of AlCl_3_ × 6H_2_O. This growth-inhibiting concentration was established earlier, assessing Al tolerance of E107 (brz) [35]. The growth-inhibiting concentration of Fe was established by preliminary experiments applying 150, 300, and 600 µM of FeCl_3_ × 6H_2_O.

Plants (6 seedlings per pot) were cultivated for 10 days in a growth chamber (ADAPTIS-A1000, Conviron, Isleham, UK) with 200 µmol of quanta m^−2^ s^−1^, a 12 h photoperiod, and minima/maxima temperatures of 18 °C/22 °C. Then, fresh biomass of individual plants (root and shoot) was determined. Three plants from each pot were individually and immediately frozen in liquid nitrogen and stored at −80 °C for further determination of phytohormones. Roots of the remaining three plants were washed for 1 min in deionized water to remove unbounded Al, Fe, and nutrients from the root surface. Then, roots or shoots of these three plants were combined, dried at 40 °C, and used to estimate the content of Al, Fe, and nutrient elements. Two independent experiments with one pot per each treatment were performed for determination of growth parameters and the content of nongaseous phytohormones and elements in plants. The third experiment with one pot per each treatment (6 plants per pot) was performed for determination of endogenous ethylene production. Then, roots or shoots of every three plants of this experiment were combined into two samples, dried at 40 °C, and used for elemental analysis.

### 4.3. Determination of Phytohormones

The frozen biomass of roots and shoots was homogenized in methanol using a RayKol AH-50 automatic homogenizer (RayKol Group (XiaMen) Corp., Ltd., Xiamen, China). The homogenate was centrifuged at 11,000 rpm and 4 °C, while the supernatant was separated and evaporated to dryness at 35 °C in a nitrogen stream using a RayKol Auto EVA-30 automatic evaporator (RayKol Group (XiaMen) Corp., Ltd., China). The resulting dry residues were dissolved in 20% acetonitrile. The samples were pre-centrifuged at 14,000 rpm and 4 °C and filtered through nylon membrane filters. The resulting solution was analyzed by HPLC-MS using an IT-TOF mass spectrometer (Shimadzu Corporation, Tokyo, Japan) equipped with an ESI interface. A Waters BEH Shield RP18 column (50 × 2.1 mm, 1.7 μm) (Waters, Milford, MA, USA) was used. Chromatography was carried out at temperature 35 °C and a flow rate of 0.3 mL min^−1^. Injection volume was 5 µL.

Concentrations of ABA, SA, and JA were analyzed by negative electrospray ionization mode. The mobile phases were 0.1% formic acid (A) and acetonitrile with 0.1% formic acid (B). The gradient was as follows: 0.0–1.0 min, 0% B, 1.0–11.0 min, 0% → 70% B; 11.0–13.0 min, 70 → 100% B; 13–15 min, 100% B and 15–15.1 min, 100% → 0% B. The IT-TOF/MS analysis was carried out in the selected mass ranges, which were (m/z): 136.5000–138.5000 Da (for SA determination); 208.5000–210.5000 Da (for JA determination); 262.5000–264.5000 Da (for ABA determination) with a scan rate 2 spectra sec^−1^. The operating parameters of the electrospray ionization sources were as follows: nebulizing gas (N_2_) flow rate, 1.5 L min^−1^; drying gas pressure, 100 kPa; CDL temperature, 200 °C; heat block temperature, 200 °C. Probe voltage was: −3.5 kV in the range of 6.0–7.6 min (for SA); −2.5 kV in the range of 7.6–8.6 min (for ABA); −1.5 kV in the range of 8.6–10.0 min (for JA). Ion accumulation time was 500 ms; detector voltage—1.6 kV. Retention times of phytohormones: JA—8.9 ± 0.3 min; ABA—7.9 ± 0.3 min; SA—6.8 ± 0.3 min. Calibration was performed using the standard reagents obtained from the Merck KGaA (Darmstadt, Germany) in the concentration range: SA—50–5000 ng ml^−1^; ABA—25–2500 ng ml^−1^; JA—100–10,000 ng ml^−1^.

Concentrations of GA3, IAA, DHZ, tZ, and tZR were analyzed by positive electrospray ionization mode. The mobile phases were 0.1% formic acid (A) and acetonitrile with 0.25% formic acid (B). The gradient was as follows: 0.0–1.0 min, 0% B, 1.0–9.0 min, 0% → 75% B; 9.0–10.0 min, 75 → 100% B; 10–11 min, 100% B and 11–11.1 min, 100% → 0% B. The IT-TOF/MS analysis was carried out in the selected mass ranges, which were (m/z): 219.9000–221.4000 Da (for *t*-zeatin determination); 352.0000–353.5000 Da (for tZR); 221.9000–223.4000 Da (for DHZ determination); 285.0000–286.5000 Da (for GA3 determination); 175.9000–176.4000 Da (for IAA determination) with a scan rate 2 spectra sec^−1^. The operating parameters of the electrospray ionization sources were as follows: nebulizing gas (N_2_) flow rate, 1.5 L min^−1^; drying gas pressure—100 kPa; CDL temperature—200 °C; heat block temperature—200 °C. Probe voltage was +1.5 kV. Ion accumulation time was 500 ms; detector voltage—1.6 kV. Retention times of phytohormones: IAA—6.0 ± 0.3 min; GA3—5.8 ± 0.3 min; tZ—4.5 ± 0.2 min; tZR—4.7 ± 0.2 min; DHZ—4.4 ± 0.2 min. Calibration was performed using the standard reagents obtained from the Merck KGaA (Darmstadt, Germany) in the concentration range: IAA—100–10,000 ng ml^−1^; GA3—10–1000 ng ml^−1^; tZ—10–1000 ng ml^−1^; tZR—10–1000 ng ml^−1^; DHZ—10–1000 ng ml^−1^. All the acquisitions and analyses of data were controlled by LabSolutions LCMSSolution software (release 3.80, Shimadzu Corporation, Tokyo, Japan). A standard solution of sodium trifluoroacetic acid (TFA) was used to calibrate the TOF–MS to increase mass accuracy.

To determine ethylene emission, roots and shoots of individual plants were placed to 40 mL glass flasks. The flasks were hermetically sealed and incubated for 1 h at 22 °C in the dark. Ethylene concentration in the gas phase was determined using gas chromatograph GC-2014 (Shimadzu Corporation, Tokyo, Japan) following the manufacturer’s instructions.

### 4.4. Elemental Analysis

The dried plants were grinded to powder and digested in a mixture of concentrated HNO_3_ and 38% H_2_O_2_ at 70 °C using the digestion system DigiBlock (LabTech, Sorisole, Italy). Content of Al, Ca, Fe, K, Mg, Mn, P, S, and Zn in the digested samples was determined using an inductively coupled plasma emission spectrometer (ICPE-9000, Shimadzu, Tokyo, Japan), according to the manufacturer’s instructions.

### 4.5. Statistical Analysis

Statistical analysis of the data was performed using the software STATISTICA version 10 (TIBCO Software Inc., Palo Alto, CA, USA). MANOVA analysis with Fisher’s LSD test and Student’s *t* test were used to evaluate differences between means. Pearson correlation and cluster analysis (standardized values, Ward’s method for linkage rules, squared Euclidian distance) were applied. Data visualization was performed using a heatmap approach implemented in R 4.4.0 with the ggplot2 (v. 3.5.2) and patchwork (v. 1.3.0) packages [93,94,95].

## 5. Conclusions

This study confirmed the reduced resistance of the E107 mutant to toxic Al and demonstrated for the first time that Fe toxicity also exerted a stronger inhibitory effect on the mutant’s growth compared to the wild-type Sparkle. These effects were most likely due to the active accumulation of these metals in the mutant’s shoots. A knock-down of *OPT3* gene modulated IAA, ethylene, GA3, tZR, DZR, and tZ contents in roots and/or shoots of plants grown under normal conditions. Significant changes in hormonal status occurred in both genotypes subjected to Al and Fe stresses. All measured groups of phytohormones in roots and shoots were involved in the response to metal toxicity. Treatment with Al-induced specific and more pronounced disturbances in phytohormone status than treatment with Fe, which was likely due to the differences in the functional role of these elements in plant metabolism. Although this article did not focus on the crosstalk between phytohormones, the known synergism and antagonism effects likely influenced their contents and plant responses to stress. It was possible that after relatively long-term exposure to toxic substances, the decrease in phytohormone content in the roots was associated with a partial loss of metabolic activity due to metal toxicity, while changes in the shoots largely reflected the plant’s defense responses. Thus, activation of the transport of toxic Al and Fe by disfunction of *OPT3* gene leads to complex metal-specific and organ-specific changes in the hormonal status of plants. Mutant E107 is a promising genetic model for studying the role of phytohormones in plant-resistance mechanisms to toxic metals.

## Figures and Tables

**Table 1:** Content of Al and Fe in cultivar Sparkle and mutant E107 cultivated in nutrient solution and treated with toxic concentrations of these metals.

Genotype and Treatments	Roots		Shoots	
	Al (µg g^−1^ DW)	Fe (µg g^−1^ DW)	Al (µg g^−1^ DW)	Fe (µg g^−1^ DW)
Sparkle				
Control	0.02 ± 0.01 a	2.3 ± 0.1 a	Nd	0.23 ± 0.01 a
80 µM AlCl_3_	2.52 ± 0.39 b	2.5 ± 0.3 ab	0.07 ± 0.02 a	0.27 ± 0.03 a
300 µM FeCl_3_	0.02 ± 0.01 a	4.7 ± 0.5 cd	Nd	0.28 ± 0.02 a
E107				
Control	0.03 ± 0.01 a	3.7 ± 0.4 bc	Nd	0.41 ± 0.05 b
80 µM AlCl_3_	4.59 ± 0.45 c	5.1 ± 0.4 d	0.22 ± 0.04 b	0.40 ± 0.06 b
300 µM FeCl_3_	0.05 ± 0.02 a	7.2 ± 0.5 e	Nd	0.73 ± 0.10 c

Means and SE of three experiments. Nd stands for not detected. Different letters indicate significant differences between genotypes and treatments (Fisher’s LSD test, *p* < 0.05, n = 4).

**Table 2:** Concentration of nutrient elements in roots of cultivar Sparkle and mutant E107 cultivated in nutrient solution and treated with toxic concentrations of Al and Fe.

Treatments	Ca (mg g^−1^ DW)	K (mg g^−1^ DW)	Mg (mg g^−1^ DW)	Mn (µg g^−1^ DW)	P (mg g^−1^ DW)	S (mg g^−1^ DW)	Zn (µg g^−1^ DW)
Sparkle							
Control	0.87 ± 0.06 ab	27.2 ± 1.1 b	1.3 ± 0.1 cd	58 ± 13 abc	16.6 ± 1.1 a	5.3 ± 0.3 c	296 ± 65 a
80 µM AlCl_3_	0.94 ± 0.05 b	**21.5 ± 1.1 a**	**0.9 ± 0.1 ab**	38 ± 8 ab	19.6 ± 0.9 a	**4.1 ± 0.3 b**	329 ± 47 a
300 µM FeCl_3_	0.82 ± 0.08 ab	**18.0 ± 1.4 a**	**0.7 ± 0.1 a**	29 ± 4 a	17.1 ± 1.2 a	**3.7 ± 0.5 ab**	415 ± 62 ab
E107							
Control	0.99 ± 0.10 b	29.3 ± 3.1 b	1.6 ± 0.1 d	107 ± 14 d	19.8 ± 0.6 a	6.3 ± 0.4 c	737 ± 93 c
80 µM AlCl_3_	**0.66 ± 0.08 a**	**18.9 ± 0.9 a**	**0.9 ± 0.1 ab**	85 ± 11 cd	18.9 ± 1.4 a	**2.9 ± 0.4 a**	667 ± 81 c
300 µM FeCl_3_	0.96 ± 0.09 b	**21.4 ± 1.7 a**	**1.2 ± 0.2 bc**	64 ± 12 bc	18.7 ± 0.8 a	**2.9 ± 0.4 a**	558 ± 83 bc

Means and SE of three experiments. Different letters indicate significant differences between genotypes and treatments (Fisher’s LSD test, *p* < 0.05, n = 4). Bold values indicate significant negative effect of treatments.

**Table 3:** Concentration of nutrient elements in shoots of cultivar Sparkle and mutant E107 cultivated in nutrient solution and treated with toxic concentrations of Al and Fe.

Treatments	Ca (mg g^−1^ DW)	K (mg g^−1^ DW)	Mg (mg g^−1^ DW)	Mn (µg g^−1^ DW)	P (mg g^−1^ DW)	S (mg g^−1^ DW)	Zn (µg g^−1^ DW)
Sparkle							
Control	2.16 ± 0.28 b	16.7 ± 0.8 a	4.3 ± 0.1 bc	100 ± 6 a	11.8 ± 0.4 a	3.0 ± 0.2 b	213 ± 17 bc
80 µM AlCl_3_	**1.25 ± 0.18 a**	18.8 ± 0.8 a	**2.8 ± 0.6 a**	80 ± 11 a	11.6 ± 0.8 a	2.5 ± 0.2 ab	199 ± 28 abc
300 µM FeCl_3_	**0.63 ± 0.06 a**	16.4 ± 1.4 a	**2.2 ± 0.3 a**	58 ± 11 a	11.1 ± 0.8 a	2.4 ± 0.1 ab	155 ± 18 ab
E107							
Control	3.91 ± 0.53 c	15.4 ± 2.4 a	5.4 ± 0.5 c	150 ± 14 b	11.7 ± 0.6 a	3.0 ± 0.3 b	260 ± 26 c
80 µM AlCl_3_	**1.05 ± 0.23 a**	15.3 ± 1.3 a	**3.4 ± 0.6 ab**	217 ± 24 c	11.4 ± 1.2 a	2.6 ± 0.3 ab	216 ± 38 bc
300 µM FeCl_3_	**0.50 ± 0.08 a**	16.5 ± 1.3 a	**2.9 ± 0.6 a**	113 ± 22 b	10.9 ± 1.1 a	**2.2 ± 0.3 a**	**135 ± 20 a**

Means and SE of three experiments. Nd stands for not detected. Different letters indicate significant differences between genotypes and treatments (Fisher’s LSD test, *p* < 0.05, n = 4). Bold values indicate significant negative effect of treatments.

## Data Availability

The raw data supporting the conclusions of this article will be made available by the authors on request.

## Supplementary Materials

The following supporting information can be downloaded at: https://www.mdpi.com/article/10.3390/plants15071129/s1, Table S1: Examples of the effects of aluminum on concentrations of phytohormones in plants; Table S2: Correlation coefficients between growth parameters and concentrations of phytohormones in plants (n = 6); Table S3: Initial data on biomass of Sparkle and E107 mutant. Table S4: Initial data on phytohormone content in roots and shoots of Sparkle; Table S5: Initial data on phytohormone content in roots and shoots of E107 mutant.
