# Supplementary material for: Hormonal Status and the Probable Role of Phytohormones in Response of Pea Cultivar Sparkle and Mutant E107 (brz) to Aluminum and Iron Toxicity
Source: Plants (Basel). 2026 Apr 7;15(7):1129. doi: 10.3390/plants15071129 (PMC13074937; doi:10.3390/plants15071129)
Supplement: Supplementary file 1 [file plants-15-01129-s001.zip › plants-4222087-Supplemental-Corrected-4.6.pdf]

SUPPLEMENTAL MATERIAL

Title of the manuscript: "**Hormonal status and the probable role of phytohormones in response of pea cultivar Sparkle and mutant E107 (brz) to aluminum and iron toxicity**".

Table S1. Examples of the effects of aluminum on concentrations of phytohormones in plants.

**Plant species**	**Treatment, μM $AlCl_3$**	**Exposition**	**Plant organ and the effect on hormones**	**Reference**
Arabidopsis thaliana	100	2 h	↓ IAA in root apices.	[21]
Arabidopsis thaliana	50	5 ÷ 25 h	↑ Ethylene in root tips.	[52]
Arabidopsis thaliana	100	10 d	↑ Total plant ACC, = ABA content.	[53]
Arabidopsis thaliana	25	3 h	↑ Local IAA accumulation in root apex.	[19]
Arabidopsis thaliana	6 ÷ 10	6 ÷ 12 h	↑ IAA in root tips.	[17]
Arabidopsis thaliana	8	7 d	↓ JA in roots.	[65]
Cassia tora	10 ÷ 50	12 h	↑ NO in root tips.	[87]
Fagopyrum esculentum	50 ÷ 100	12 ÷ 72 h	↑ ABA in roots.	[62]
Glycine max	30 ÷ 50	3 ÷ 12 h	↑ ABA in roots and leaves.	[61]
Glycine max	30	12 h	↑ Free and conjugated SA in root tips.	[74]
Glycine max	30	2 ÷ 9 h	↑ SA in roots.	[73]
Hordeum vulgare	200 and 300	72 h	↑ ABA in leaves.	[58]
Hordeum vulgare	100	24 h	↑ ABA in roots.	[60]
Lotus japonicus	50	2 and 24 h	↑ Ethylene in root apices.	[51]
Medicago sativa	100	1, 3 and 10 d	↓ IAA in root tips and apical buds.	[22]
Oryza sativa	500	24 ÷ 72 h	↑ ABA and JA in roots.	[59]
Oryza sativa	25	3 d	↑ IAA in root apices.	[18]
Oryza sativa	50	24 h	↑ IAA in root tips.	[16]
Phaseolus vulgaris	127	120 h	↑ ZR and DHZR in roots, stems and leaves.	[23]
Phaseolus vulgaris	127	25 ÷ 150 min	↑ Ethylene, Z, ZMP and DHZR in root tips.	[24]
Picea abies	5600	0.5 and 40 h	↑ IAA and GAs, ↓ CKs in root tips.	[14]
Triticum aestivum	50 and 200	2 h	↑ Ethylene in root apices.	[54]
Triticum aestivum	25, 50 and 100	24 h	↑ IAA in root apices.	[15]
Zea mays	90	1 h	↓ IAA transport in the root elongation zone.	[20]

All experiments were conducted using hydroponic cultures. Notes: ↑ - increase, ↓ - decrease, = - no effect.
Abbreviations: ABA - abscisic acid, ACC - 1-aminocyclopropane-1-carboxylic acid, Al - aluminium, CKs - cytokinins, DHZR - dihydrozeatin riboside, GAs - gibberellins, IAA – indole-3-acetic acid, NO – nitric oxide, SA- salicylic acid, Z - zeatin, ZMP - zeatin riboside-5-monophosphate, ZR - zeatin riboside.

References cited in Table S1:

14. Čížková, R. Phytohormonal levels in spruce roots under aluminium stress. In *Structure and Function of Roots: Developments in Plant and Soil Sciences*; Baluška, F., Čiamporová, M., Gašparíková, O., Barlow P.W., Eds.; Springer: Dordrecht, Netherlands, 1995; Volume 58, pp. 335–339. https://doi.org/10.1007/978-94-017-3101-0_44.
15. Yang, Y.; Wang, Q.; Geng, M.; Guo, Z.; Zhao, Z. Effect of indole-3-acetic acid on aluminum-induced efflux of malic acid from wheat (*Triticum aestivum* L.). *Plant Soil* **2011**, *346*, 215–230. https://doi.org/10.1007/s11104-011-0811-1.
16. Wu, D.; Shen, H.; Yokawa, K.; Baluška, F. Alleviation of aluminium-induced cell rigidity by overexpression of *OsPIN2* in rice roots. *J. Exp. Bot.* **2014**, *65*, 5305–5315. https://doi.org/10.1093/jxb/eru292.
17. Yang, Z.-B.; Geng, X.; He, C.; Zhang, F.; Wang, R.; Horst, W.J.; Ding, Z. TAA1-regulated local auxin biosynthesis in the root-apex transition zone mediates the aluminum-induced inhibition of root growth in *Arabidopsis*. *Plant Cell* **2014**, *26*, 2889–2904. https://doi.org/10.1105/tpc.114.127993.
18. Wang, M.; Qiao, J.; Yu, C.; Chen, H.; Sun, C.; Huang, L.; Li, C.; Geisler, M.; Qian, Q.; Jiang, A.; Qi, Y. The auxin influx carrier, OsAUX3, regulates rice root development and responses to aluminium stress. *Plant Cell Environ.* **2019**, *42*, 1125–1138. https://doi.org/10.1111/pce.13478.
19. Li, C.; Liu, G.; Geng, X.; He, C.; Quan, T.; Hayashi, K.I.; De Smet, I.; Robert, H.S.; Ding, Z.; Yang, Z.B. Local regulation of auxin transport in root-apex transition zone mediates aluminium-induced *Arabidopsis* root-growth inhibition. *Plant J.* **2021**, *108*, 55–66. https://doi.org/10.1111/tpj.15424.
20. Kollmeier, M.; Felle, H.H.; Horst, W.J. Genotypical differences in aluminum resistance of maize are expressed in the distal part of the transition zone. Is reduced basipetal auxin flow involved in inhibition of root elongation by aluminum? *Plant Physiol.* **2000**, *122*, 945–956. https://doi.org/10.1104/pp.122.3.945.
21. Shen, H.; Hou, N.; Schlicht, M.; Wan, Y.; Mancuso, S.; Baluska, F. Aluminium toxicity targets PIN2 in *Arabidopsis* root apices: effects on PIN2 endocytosis, vesicular recycling, and polar auxin transport. *Chin. Sci. Bull.* **2008**, *53*, 2480–2487. https://doi.org/10.1007/s11434-008-0332-3.
22. Wang, S.; Yuan, S.; Su, L.; Lv, A.; Zhou, P.; An, Y. Aluminum toxicity in alfalfa (*Medicago sativa*) is alleviated by exogenous foliar IAA inducing reduction of Al accumulation in cell wall. *Environ. Exp. Bot.* **2017**, *139*, 1–13. https://doi.org/10.1016/j.envexpbot.2017.03.018.
23. Massot, N.; Poschenrieder, Ch.; Barcelo, J. Aluminium-induced increase of zeatin riboside and dihydrozeatin riboside in *Phaseolus vulgaris* L. cultivars. *J. Plant Nutr.* **1994**, *17*, 255–265. https://doi.org/10.1080/01904169409364725.
24. Massot, N.; Nicander, B.; Barcelo, J.; Poschenrieder, C.; Tillberg, E. A rapid increase in cytokinin levels and enhanced ethylene evolution precede Al^{3+}-induced inhibition of root growth in bean seedlings (*Phaseolus vulgaris* L.). *Plant Growth Regul.* **2002**, *37*, 105–112. https://doi.org/10.1023/A:1020511007138.
51. Sun, P.; Tian, Q.-Y.; Zhao, M.-G.; Dai, X.-Y.; Huang, J.-H.; Li, L.-H; Zhang, W.-H. Aluminum-induced ethylene production is associated with inhibition of root elongation in *Lotus japonicus* L. *Plant Cell Physiol.* **2007**, *48*, 1229–1235. https://doi.org/10.1093/pcp/pcm077.
52. Sun, P.; Tian, Q.Y.; Chen, J.; Zhang, W.H. Aluminium-induced inhibition of root elongation in *Arabidopsis* is mediated by ethylene and auxin. *J. Exp. Bot.* **2010**, *61*, 347–356. https://doi.org/10.1093/jxb/erp306.
53. Li, L.; Li, X.; Yang, C.; Cheng, Y.; Cai, Z.; Nian, H.; Ma, Q. *GsERF1* enhances *Arabidopsis thaliana* aluminum tolerance through an ethylene-mediated pathway. *BMC Plant Biol.* **2022**, *22*, 258 https://doi.org/10.1186/s12870-022-03625-6.
54. Tian, Q.; Zhang, X.; Ramesh, S.; Gilliham, M.; Tyerman, S.D.; Zhang, W.H. Ethylene negatively regulates aluminium-induced malate efflux from wheat roots and tobacco cells transformed with *TaALMT1*. *J. Exp. Bot.* **2014**, *65*, 2415–2426. https://doi.org/10.1093/jxb/eru123.
58. Klimashevskii, E.L.; Chernysheva, N.F. Content of organic acids and physiologically active substances in plants which differ in sensitivity to Al^{3+} toxicity. *Soviet Agricultural Sciences* **1980**, *2*, 5–7.
59. Roselló, M.; Poschenrieder, C.; Gunsé, B.; Barceló, J.; Llugany, M. Differential activation of genes related to aluminium tolerance in two contrasting rice cultivars. *J. Inorg. Biochem.* **2015**, *152*, 160–6. https://doi.org/10.1016/j.jinorgbio.2015.08.021.
60. Kasai, M.; Sasaki, M.; Tanakamaru, S.; Yamamoto, Y.; Matsumoto H. Possible involvement of abscisic acid in increases in activities of two vacuolar H^+-pumps in barley roots under aluminum stress. *Plant Cell Physiol.* **1993**, *34*, 1335–1338. https://doi.org/10.1093/oxfordjournals.pcp.a078558.

61. Hou, N.; You, J.; Pang, J.; Xu M.; Chen G.; Yang Z.M. The accumulation and transport of abscisic acid insoybean (*Glycine max* L.) under aluminum stress. *Plant Soil* **2010**, *330*, 127–137. https://doi.org/10.1007/s11104-009-0184-x.
62. Reyna-Llorens, I.; Corrales, I.; Poschenrieder, C.; Barcelo, J.; Cruz-Ortega, R. Both aluminum and ABA induce the expression of an ABC-like transporter gene (*FeALS3*) in the Al-tolerant species *Fagopyrum esculentum*. Environ. Exp. Bot. **2015**, *111*, 74–82. https://doi.org/10.1016/j.envexpbot.2014.11.005.
65. Yang, Z.B.; Liu, G.; Liu, J.; Zhang, B.; Meng, W.; Müller, B.; Hayashi, K.I.; Zhang, X.; Zhao, Z.; De Smet, I.; Ding, Z. Synergistic action of auxin and cytokinin mediates aluminum-induced root growth inhibition in *Arabidopsis*. *EMBO Rep.* **2017**, *18*, 1213–1230. https://doi.org/10.15252/embr.201643806.
73. Liu, N.; You, J.; Shi, W.; Liu W. Yang Z. Salicylic acid involved in the process of aluminum induced citrate exudation in *Glycine max* L. *Plant Soil* **2012**, *352*, 85–97. https://doi.org/10.1007/s11104-011-0981-x.
74. Lan, T.; You, J.; Kong, L.; Yu, M.; Liu, M.; Yang, Z. The interaction of salicylic acid and Ca(2+) alleviates aluminum toxicity in soybean (Glycine max L.). *Plant Physiol. Biochem.* **2016**, *98*, 146–54. https://doi.org/10.1016/j.plaphy.2015.11.019.
87. Xue, Y.J.; Tao, L.; Yang, Z.M. Aluminum-induced cell wall peroxidase activity and lignin synthesis are differentially regulated by jasmonate and nitric oxide. *J. Agric. Food. Chem.* **2008**, *56*, 9676–9684. https://doi.org/10.1021/jf802001v.

Table S2. Correlation coefficients between growth parameters and concentrations of phytohormones in plants (n = 6).

	Shoot FW	Root ABA	Root SA	Root JA	Root DHZ	Root tZ	Root tZR	Root GA3	Root Eth	Root IAA	Shoot ABA	Shoot SA	Shoot JA	Shoot DHZ	Shoot tZ	Shoot tZR	Shoot GA3	Shoot Eth	Shoot IAA
**Root FW**	**0.92**	0.57	0.48	**−0.88**	**−0.83**	0.34	−0.35	−0.31	0.07	0.31	**−0.97**	**−0.83**	−0.22	−0.60	−0.71	0.14	−0.80	0.38	−0.80
	p=0.010	p=0.234	p=0.338	p=0.022	p=0.040	p=0.510	p=0.502	p=0.554	p=0.897	p=0.554	p=0.001	p=0.049	p=0.676	p=0.209	p=0.113	p=0.795	p=0.055	p=0.459	p=0.051
**Shoot FW**		0.60	0.20	−0.69	−0.70	0.57	−0.05	−0.16	0.28	0.27	**−0.90**	−0.60	−0.66	−0.39	−0.38	−0.022	−0.67	0.62	−0.52
		p=0.210	p=0.707	p=0.130	p=0.119	p=0.237	p=0.926	p=0.762	p=0.592	p=0.605	p=0.015	p=0.211	p=0.156	p=0.444	p=0.464	p=0.967	p=0.147	p=0.186	p=0.287
**Root ABA**			0.15	−0.28	−0.33	0.38	0.23	−0.48	−0.43	−0.06	−0.49	−0.08	**−0.91**	0.30	0.24	0.28	−0.21	0.57	0.30
			p=0.779	p=0.594	p=0.517	p=0.456	p=0.666	p=0.339	p=0.393	p=0.916	p=0.318	p=0.875	p=0.010	p=0.560	p=0.641	p=0.587	p=0.684	p=0.235	p=0.563
**Root SA**				−0.78	−0.59	0.03	−0.27	−0.77	−0.08	0.06	−0.59	−0.44	0.12	−0.36	−0.75	0.39	−0.73	0.058	−0.40
				p=0.065	p=0.217	p=0.955	p=0.599	p=0.074	p=0.873	p=0.906	p=0.214	p=0.380	p=0.813	p=0.479	p=0.085	p=0.439	p=0.098	p=0.914	p=0.434
**Root JA**					0.78	−0.29	0.47	0.51	−0.11	−0.41	**0.92**	0.81	0.10	0.50	**0.82**	−0.14	**0.87**	−0.20	0.74
					p=0.066	p=0.577	p=0.346	p=0.295	p=0.842	p=0.420	p=0.010	p=0.050	p=0.856	p=0.306	p=0.047	p=0.796	p=0.022	p=0.698	p=0.090
**Root DHZ**						0.03	0.24	0.17	−0.29	0.16	**0.88**	0.76	0.29	0.74	0.64	−0.57	**0.96**	−0.69	0.63
						p=0.954	p=0.652	p=0.742	p=0.583	p=0.760	p=0.022	p=0.080	p=0.574	p=0.090	p=0.173	p=0.236	p=0.003	p=0.131	p=0.179
**Root tZ**							0.38	−0.42	0.25	0.48	−0.40	0.11	−0.47	0.089	−0.18	−0.65	−0.11	0.10	−0.13
							p=0.454	p=0.408	p=0.630	p=0.329	p=0.435	p=0.836	p=0.345	p=0.867	p=0.733	p=0.166	p=0.841	p=0.855	p=0.803
**Root tZR**								−0.09	0.29	−0.55	0.23	0.78	−0.47	0.11	0.33	−0.05	0.23	0.43	0.52
								p=0.865	p=0.572	p=0.255	p=0.654	p=0.070	p=0.348	p=0.835	p=0.517	p=0.926	p=0.666	p=0.399	p=0.287
**Root GA3**									0.37	−0.17	0.39	−0.01	0.21	−0.19	0.33	−0.09	0.32	0.04	−0.08
									p=0.474	p=0.740	p=0.443	p=0.983	p=0.688	p=0.715	p=0.519	p=0.860	p=0.534	p=0.946	p=0.873
**Root Eth**										−0.13	−0.22	−0.08	0.12	−0.79	−0.50	−0.23	−0.38	0.32	−0.59
										p=0.803	p=0.675	p=0.882	p=0.817	p=0.063	p=0.312	p=0.665	p=0.460	p=0.534	p=0.222
**Root IAA**											−0.24	−0.38	0.18	0.21	−0.26	−0.72	0.03	−0.58	−0.41
											p=0.649	p=0.455	p=0.727	p=0.693	p=0.618	p=0.106	p=0.948	p=0.226	p=0.416
**Shoot ABA**												0.75	0.43	0.54	0.66	−0.19	**0.98**	−0.53	0.66
												p=0.084	p=0.399	p=0.265	p=0.149	p=0.715	p=0.015	p=0.274	p=0.150
**Shoot SA**													−0.051	0.54	0.61	−0.22	0.73	−0.16	0.80
													p=0.924	p=0.267	p=0.195	p=0.671	p=0.100	p=0.755	p=0.055
**Shoot JA**														−0.17	−0.32	−0.17	0.15	−0.74	−0.29
														p=0.741	p=0.534	p=0.753	p=0.773	p=0.095	p=0.581
**Shoot DHZ**															0.77	−0.27	0.79	−0.45	0. 80
															p=0.075	p=0.607	p=0.060	p=0.368	p=0.057
**Shoot tZ**																−0.02	**0.82**	−0.04	**0.86**
																p=0.965	p=0.044	p=0.934	p=0.027
**Shoot tZR**																	−0.42	0.55	.12
																	p=0.408	p=0.262	p=0.821
**Shoot GA3**																		−0.54	0.73
																		p=0.266	p=0.097
**Shoot Eth**																			−0.06
																			p=0.903

Red and **blue** bold values indicate significant positive and negative correlation, respectively.

Table S3. Initial data on biomass of Sparkle and E107 mutant.

Treatments	Sparkle		E107	
	Root (g FW plant^{-1})	Shoot (g FW plant^{-1})	Root (g FW plant^{-1})	Shoot (g FW plant^{-1})
Contol	0.830	0.775	0.720	0.870
Contol	1.100	0.690	0.930	0.935
Contol	0.870	0.825	1.030	1.075
Contol	0.845	0.795	0.930	0.935
Contol	1.245	0.965	1.100	0.425
Contol	0.970	0.870	1.070	0.415
Contol	0.950	0.718	0.465	0.503
Contol	0.711	0.668	0.485	0.662
Contol	0.711	0.620	0.741	0.640
Contol	0.655	0.513	0.786	0.914
Contol	0.884	0.643	0.678	0.625
Contol	0.682	0.770	0.519	0.612
Al	0.805	0.865	0.612	0.820
Al	0.795	0.755	0.546	0.740
Al	0.925	0.770	0.606	0.830
Al	0.880	0.535	0.885	0.720
Al	0.850	0.800	0.708	0.390
Al	0.757	0.873	0.720	0.362
Al	0.483	0.407	0.645	0.342
Al	0.472	0.308	0.440	0.647
Al	0.989	0.694	0.840	0.584
Al	0.954	0.734	0.456	0.281
Al	0.536	0.639	0.444	0.552
Al	0.844	0.753	0.344	0.507
Fe	1.075	0.620	0.870	0.605
Fe	1.000	0.450	0.730	0.610
Fe	0.910	0.600	0.800	0.625
Fe	1.100	0.690	0.975	0.675
Fe	0.990	0.765	0.650	0.775
Fe	0.910	0.717	0.448	0.684
Fe	0.920	0.713	0.429	0.361
Fe	0.668	0.688	0.644	0.391
Fe	0.723	0.729	0.411	0.341
Fe	0.594	0.573	0.429	0.310
Fe	0.434	0.576	0.448	0.371
Fe	0.431	0.831	0.406	0.565

Table S4. Initial data on phytohormone content in roots and shoots of Sparkle.
Data are given as ng g^{-1} FW for all phytohormones except for ethylene as pmol h^{-1} g^{-1} FW.

Treatments	Roots									Shoots								
	ABA	SA	JA	DHZ	tZ	tZR	GA3	C_2H_4	IAA	ABA	SA	JA	DHZ	tZ	tZR	GA3	C_2H_4	IAA
Control	1.24	840	2.0	0.62	3.58	0.11	0.019	14.6	367	4.4	3.4	8.6	0.17	17.67	0.25	0.04	21.8	13.7
Control	1.70	647	4.0	0.52	2.10	0.21	0.012	27.7	354	3.1	2.3	7.2	0.60	20.11	0.30	0.04	36.4	15.0
Control	1.47	409	6.3	0.89	1.47	0.23	0.012	21.1	408	3.1	3.7	7.5	0.6	21.1	0.3	0.0	18.6	22.7
Control	2.45	989	5.3	1.27	1.82	0.26	0.007	25.1	434	2.8	1.1	6.7	1.15	23.93	0.44	0.01	16.1	14.2
Control	2.04	386	3.7	0.79	1.71	0.38	0.010	18.9	354	2.7	2.8	12.4	0.62	34.97	0.28	0.04	33.6	11.3
Control	1.72	610	2.1	0.39	2.33	0.05	0.014	15.3	207	3.9	1.8	4.1	0.42	16.85	0.35	0.06	21.6	10.6
Al	1.07	89	10.2	1.04	0.83	0.63	0.045	31.6	174	5.5	3.6	14.8	0.06	26.35	1.98	0.03	27.6	17.8
Al	1.06	85	8.7	0.34	0.63	0.81	0.034	18.8	262	6.5	2.7	11.5	0.3	28.2	1.1	0.0	37.8	21.6
Al	1.46	66	2.3	0.25	0.41	0.28	0.039	25.6	270	9.2	4.3	17.0	0.29	28.42	1.67	0.04	27.7	13.8
Al	1.80	97	10.0	0.59	0.61	0.60	0.021	35.6	262	8.8	2.6	10.8	0.15	38.55	0.51	0.05	31.2	14.3
Al	1.18	80	8.2	0.49	0.59	0.54	0.025	36.2	389	6.4	1.6	7.2	0.08	24.50	0.47	0.01	33.2	12.8
Al	0.52	62	9.8	0.22	0.47	0.38	0.005	38.1	229	5.7	1.0	5.9	0.38	26.15	1.48	0.04	33.3	8.2
Fe	2.39	1676	0.1	0.12	0.12	0.47	0.005	17.8	237	2.6	2.3	17.7	0.20	15.01	2.34	0.01	19.8	8.9
Fe	2.26	1596	1.7	0.24	0.24	0.48	0.004	25.1	275	6.1	1.6	22.9	0.19	14.52	2.90	0.01	30.9	9.5
Fe	1.34	1522	3.8	0.29	0.67	0.41	0.008	14.3	259	3.8	0.4	11.3	0.23	19.65	2.30	0.02	37.6	32.3
Fe	1.50	1646	4.2	0.36	1.77	0.66	0.008	14.3	268	4.1	6.3	3.0	0.64	14.30	0.89	0.02	16.7	18.6
Fe	0.80	1478	7.7	0.39	0.97	0.18	0.010	23.4	346	4.1	2.7	13.7	0.3	14.3	1.8	0.0	38.5	22.6
Fe	1.16	1214	4.9	0.38	0.23	0.25	0.014	21.5	208	4.1	2.6	13.7	0.32	6.35	0.18	0.01	29.9	17.0

Table S5. Initial data on phytohormone content in roots and shoots of E107 mutant.
Data are given as ng g^{-1} FW for all phytohormones except for ethylene as pmol h^{-1} g^{-1} FW.

**Treatments**	**Roots**									**Shoots**								
	**ABA**	**SA**	**JA**	**DHZ**	**tZ**	**tZR**	**GA3**	**C_2H_4**	**IAA**	**ABA**	**SA**	**JA**	**DHZ**	**tZ**	**tZR**	**GA3**	**C_2H_4**	**IAA**
Control	1.48	1152	1.1	0.70	2.47	1.77	0.004	43.9	300	5.4	4.3	9.0	0.21	7.61	0.34	0.01	37.5	9.7
Control	1.71	595	8.9	0.84	2.95	2.05	0.027	38.2	275	2.3	5.5	13.3	0.00	13.97	0.38	0.01	33.4	10.0
Control	1.06	753	1.3	0.57	2.07	2.07	0.013	56.4	248	4.1	2.7	2.6	0.14	13.81	0.90	0.03	42.0	6.5
Control	1.83	782	5.2	0.14	2.31	1.00	0.010	40.9	300	3.8	3.5	8.1	0.1	11.2	0.6	0.0	13.3	21.5
Control	1.37	819	5.5	0.52	2.31	1.69	0.012	31.6	256	4.1	2.4	6.5	0.25	11.57	1.16	0.04	48.8	18.5
Control	0.73	813	11	0.28	1.73	1.33	0.011	22.2	276	2.9	2.3	7.6	0.08	9.49	0.51	0.01	17.9	13.8
Al	1.07	37	10.0	1.58	1.11	1.28	0.016	20.0	159	6.7	4.1	9.4	0.70	34.20	0.57	0.04	18.3	27.8
Al	1.64	62	12.7	1.36	1.30	1.63	0.017	17.4	258	12.2	4.3	6.9	0.8	43.6	0.8	0.1	30.6	31.1
Al	2.46	42	8.3	1.20	1.20	0.75	0.014	14.3	206	14.0	5.1	2.3	0.81	45.03	1.12	0.11	38.1	52.6
Al	1.56	55	16.5	1.45	0.83	1.91	0.004	24.5	435	10.8	7.5	2.4	0.97	52.95	0.75	0.11	30.3	54.8
Al	1.00	90	20.1	1.71	1.24	2.18	0.031	20.7	248	22.9	4.9	6.0	1.03	72.39	0.94	0.04	22.6	23.6
Al	2.09	85	7.4	0.77	2.13	1.90	0.012	10.6	252	14.8	1.3	13.4	0.72	40.47	0.98	0.10	20.5	39.9
Fe	1.06	733	12.2	0.91	0.78	0.36	0.015	19.9	356	5.8	2.9	41.8	0.45	22.60	0.55	0.04	18.8	22.6
Fe	0.10	849	10.3	2.10	1.32	0.48	0.044	25.4	432	14.0	3.8	30.4	0.79	19.58	0.72	0.04	19.2	7.5
Fe	0.10	440	9.1	0.61	0.90	0.36	0.007	26.9	315	12.8	3.3	28.5	0.5	21.4	0.5	0.1	12.2	13.7
Fe	0.12	527	9.4	1.27	0.93	0.39	0.020	39.8	278	21.2	2.6	24.2	0.37	15.68	0.06	0.08	18.4	15.0
Fe	0.04	510	8.8	1.45	1.01	0.46	0.006	32.7	308	11.3	1.9	28.7	0.06	18.03	0.13	0.05	22.9	17.8
Fe	0.28	276	2.7	0.94	0.58	0.23	0.024	27.2	175	9.9	5.1	22.6	0.29	13.26	0.46	0.07	17.4	16.9
